# Supplementary material for: Detection of selection signatures in farmed coho salmon (Oncorhynchus kisutch) using dense genome-wide information
Source: Sci Rep. 2021 May 6;11:9685. doi: 10.1038/s41598-021-86154-w (PMC8102513; doi:10.1038/s41598-021-86154-w)
Supplement: Supplementary file 1 — Supplementary Information 1. [file 41598_2021_86154_MOESM1_ESM.docx]

**Detection of selection signatures in farmed coho salmon (*Oncorhynchus kisutch*) using dense genome-wide information**

López M.E.^a^, Cádiz M.I.^b^, Rondeau E.B.^c^, Koop B.F.^c^, Yáñez J.M.^b,d*^

^a^ Department of Aquatic Resources, Swedish University of Agricultural Sciences, Drottningholm, Sweden.

^b^ Facultad de Ciencias Veterinarias y Pecuarias, Universidad de Chile, Santiago, Chile.

^c^ Department of Biology, University of Victoria, British Columbia, Canada.

^d^ Núcleo Milenio INVASAL, Concepción, Chile

^*^jmayanez@uchile.cl +56-2 29785533 (Corresponding Author).

**Supplementary information**

**Supplementary Table S1.** Regions with at least two SNPs above the threshold, detected by iHS. Definition of columns: **(1)** Chromosome **(2)** Position of the first SNP **(3)** Position of the last SNP **(4)** Start of the region (First SNP minus 250 kb) **(5)** End of the region (Last SNP plus 250 kb) **(6)** Region name **(7)** Maximum –log_10_(*p*-value) in the region **(8)** Maximum |iHS| in the region **(9)** Number of SNPs in the region **(10)** Population **(11)** FDR adjustment.

**Supplementary Table S2.** Regions with at least two SNPs above the threshold, detected by XP-EHH. Definition of columns: **(1)** Chromosome **(2)** Position of the first SNP **(3)** Position of the last SNP **(4)** Start of the region (First SNP minus 250 kb) **(5)** End of the region (Last SNP plus 250 kb) **(6)** Region name **(7)** Maximum –log_10_(*p*-value) in the region **(8)** Maximum XP-EHH in the region **(9)** Number of SNPs in the region **(10)** Population **(11)** FDR adjustment.

**Supplementary Table S3.** Regions above the threshold, detected by CLR. Definition of columns: **(1)** Chromosome **(2)** Start of the region (First SNP minus 250 kb) **(3)** End of the region (Last SNP plus 250 kb) **(4)** Region name **(5)** ALPHA value in the region **(6)** CLR score in the region **(7)** Population.

**Supplementary Table S4.** Genes identified by iHS, XP-EHH and CLR. Definition of columns: **(1)** Chromosome **(2)** Start of the region **(3)** End of the region **(4)** Population **(5)** Test **(6)** Gene Name in Coho salmon **(7)** Gene Name in zebra fish.

**Supplementary Table S5.** Gene Ontology (GO) terms and KEGG (Kyoto Encyclopedia of Genes and Genomes) pathways identified in this study based on iHS, XP-EHH, CLR results.

**Supplementary Figure S1.** **.** Decay of linkage disequilibrium (LD) by chromosome for each population. Different color lines represent populations: Pop-A=green, Pop-B = magenta.

**Supplementary Figure S2.** Cross-validation error for ADMIXTURE results calculated for K values from 1 to 20.

**Supplementary Figure S3.** Individual assignment probabilities generated with ADMIXTURE (2⩽K⩽11). Each color represents a cluster, and the ratio of vertical lines is proportional to assignment probability of an individual to each cluster.
