# Supplementary figures and images for: Detection of selection signatures in farmed coho salmon (Oncorhynchus kisutch) using dense genome-wide information
Source: Sci Rep. 2021 May 6;11:9685. doi: 10.1038/s41598-021-86154-w (PMC8102513; doi:10.1038/s41598-021-86154-w)

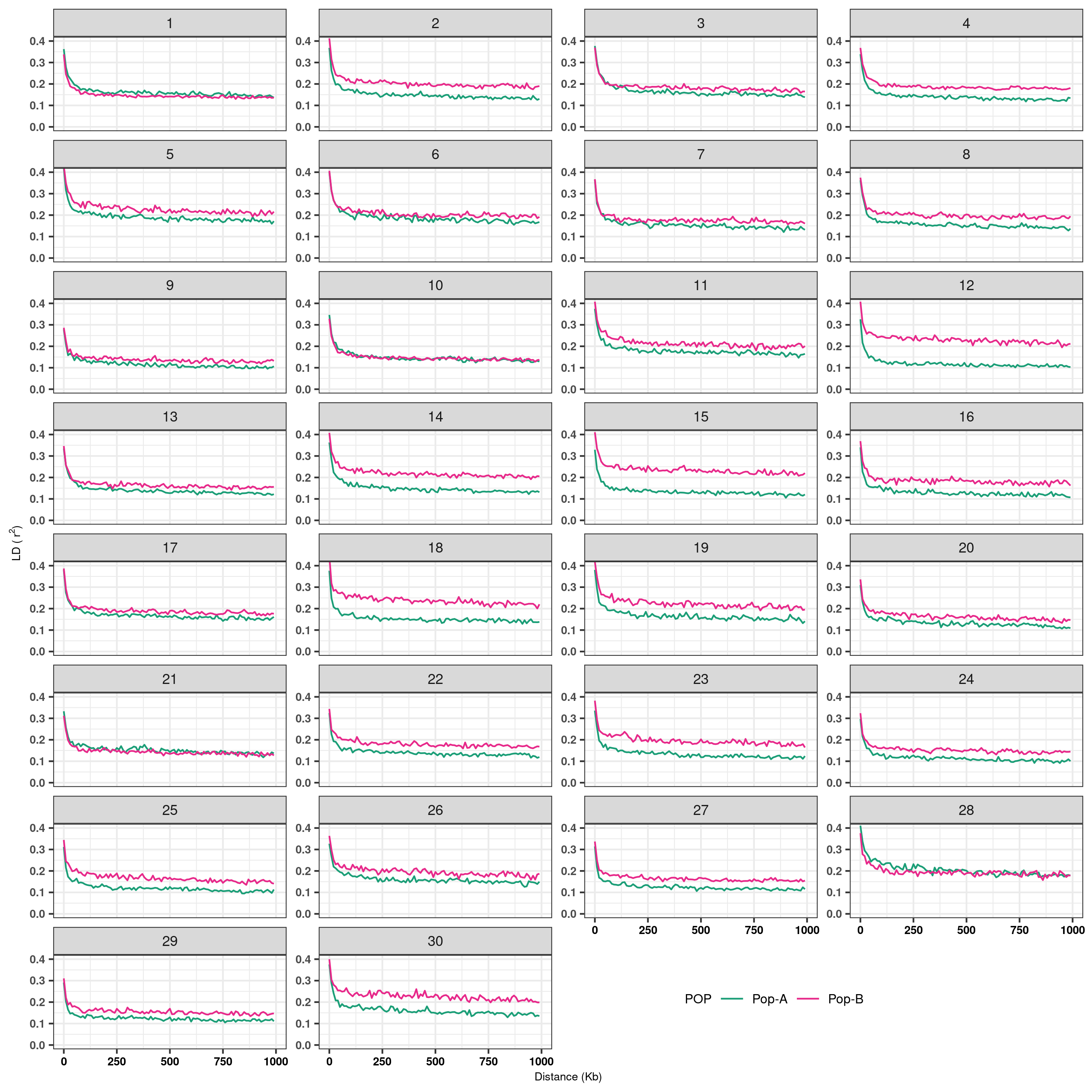

Supplement: Supplementary file 2 — Supplementary Information 2. [file 41598_2021_86154_MOESM2_ESM.tiff]

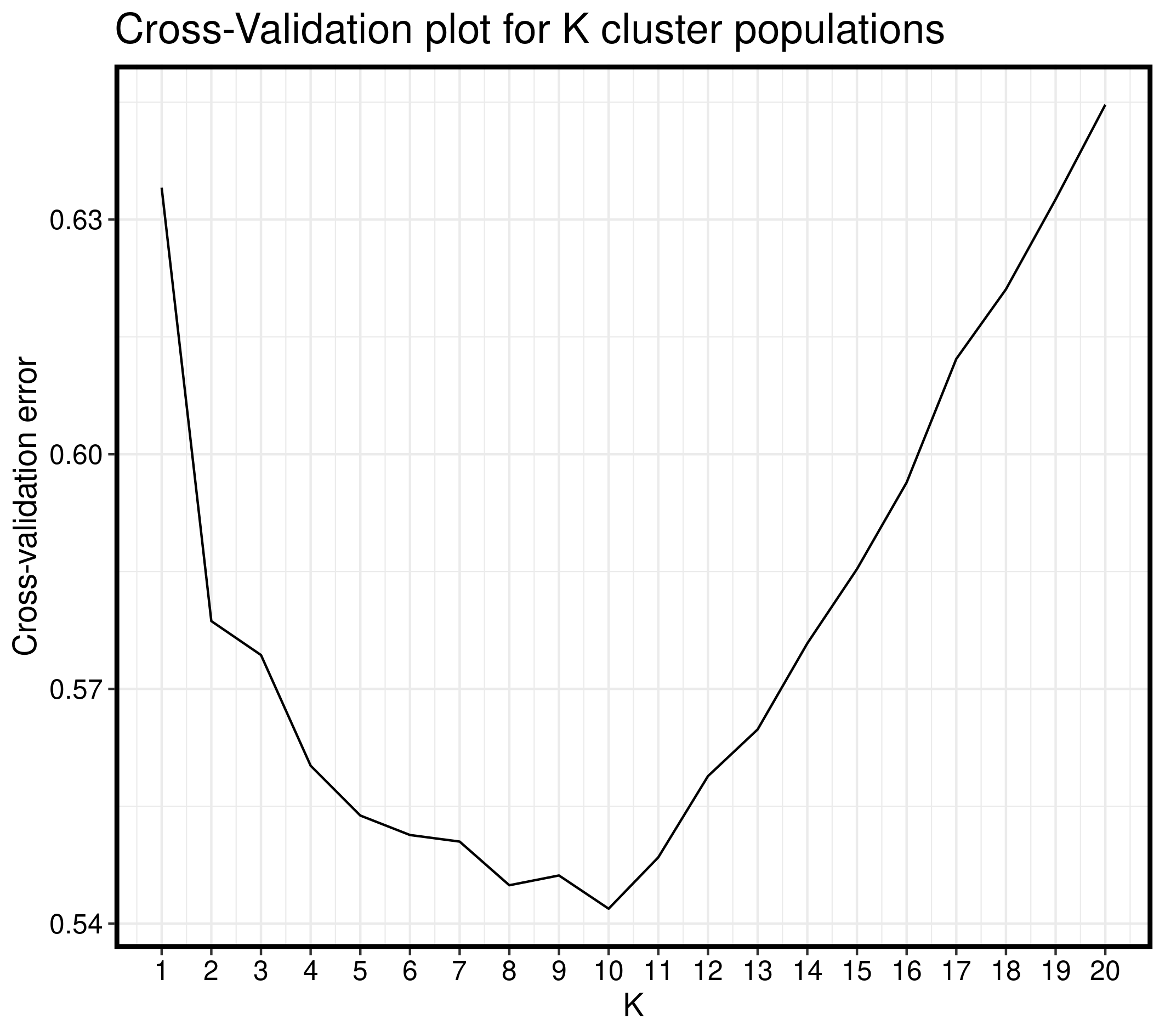

Supplement: Supplementary file 3 — Supplementary Information 3. [file 41598_2021_86154_MOESM3_ESM.tiff]

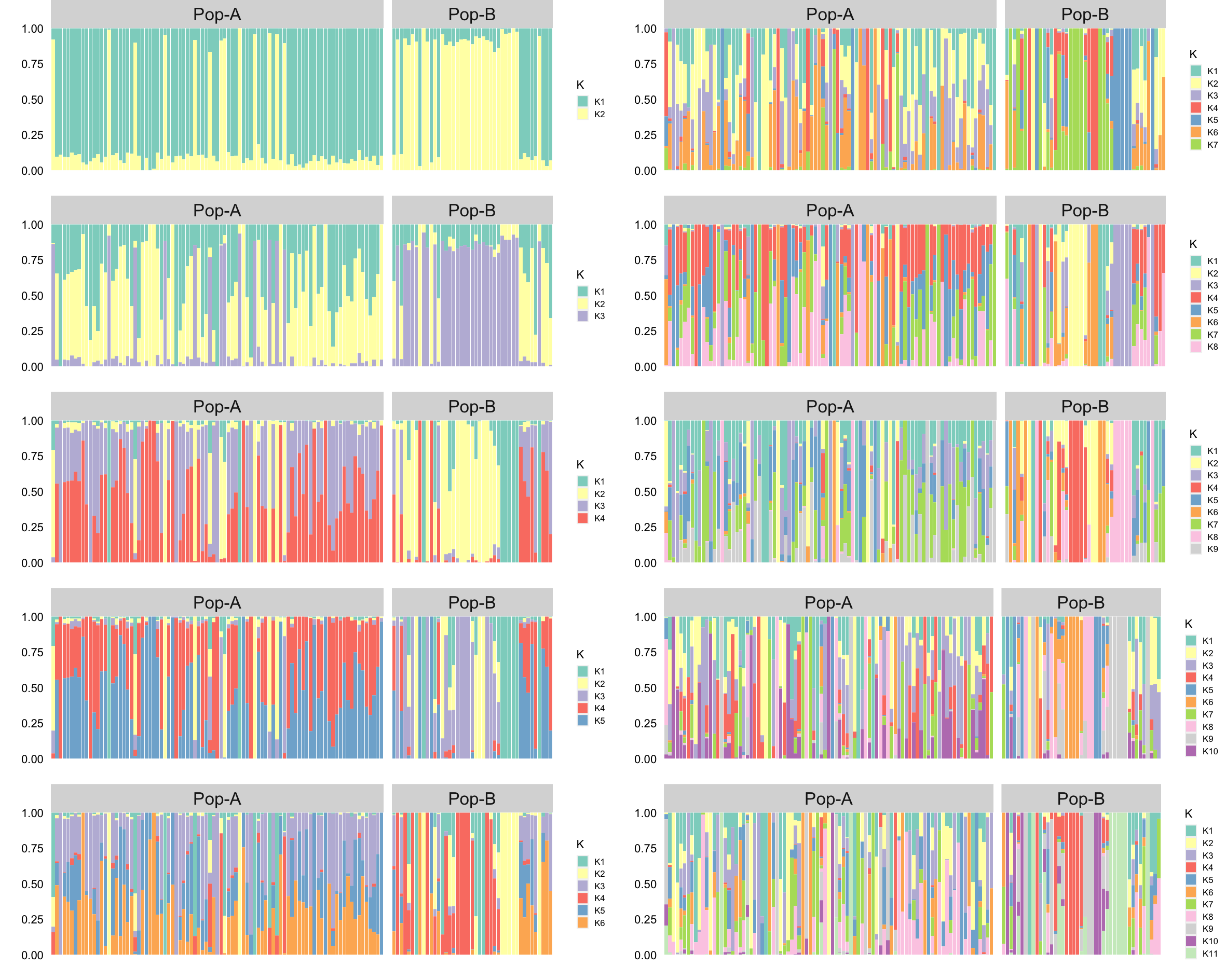

Supplement: Supplementary file 4 — Supplementary Information 4. [file 41598_2021_86154_MOESM4_ESM.tiff]
